# The Curriculum Reform of Design Education Based on the Orientation of Positive Psychology

**DOI:** 10.3389/fpsyg.2022.836909

**Published:** 2022-04-07

**Authors:** Yi Wu, Kymn Kyungsun

**Affiliations:** School of Fine Arts, Seoul National University, Seoul, South Korea

**Keywords:** positive psychology, design education, curriculum reform, applied research, cultivation grit

## Abstract

In the process of China’s rapid development, the society has higher and higher requirements for educational reform. Different from other basic disciplines, design emphasizes practicality, which requires that in the process of design education reform, more attention should be paid to the stimulation of students’ subjective initiative and the improvement of students’ ability to solve problems in the face of setbacks. This paper methodically expounds on a more scientific manner of curriculum reform fit for China’s educational system, based on positive psychology and consideration of grit. It performs research and analysis on the design education curriculum reform on the basis of positive psychology through field research, logical analysis, and other research methodologies. Designing education curriculum reform based on positive psychology orientation is more feasible than standard education curriculum reform and the role of grit in taking part in the educational curriculum reform oriented to positive psychology is considerably higher than that in the conventional educational curriculum reform, according to experimental study.

## Introduction

Nowadays, in China, several issues and social contradictions have emerged as a result of rapid development. As a result, many scholars refer to the current stage of Chinese society as a transitional period. During a country’s or nation’s transition period, its education system will often undergo various changes and transformations. Because of the advancement and foresight of modern education’s development, educational reform and transformation must come before societal reform and transformation, and educational development’s advancement and foresight must be ensured by educational theories and thoughts’ advancement and foresight. Since the last few years, with the country’s attention and the ongoing changes in the society’s need for talents, colleges and universities have conducted numerous investigations on changing the curriculum and teaching content system (Tadesse and Melese, 2016; Yun, 2017). However, the result falls well short of society’s actual demands (Shmuel, 2018; Campbel, 2020). Positive psychology maintains that only by cultivating and increasing positive power, human nature can be eradicated or suppressed, and as a new trend in psychology, the positive educational idea supported by positive psychology gives a new study viewpoint. Although most colleges and universities’ talent development goals are all positioned at “technical application talents,” their curriculum settings and teaching methods are not in keeping with the name (Koh, 2017; Mohamed and Ahmad, 2019). Many academics have had limited success with positive psychology studies. For instance, Li Jinzhen et al. in “Positive Psychology: A New Research Direction” explored the origins, basic research topics, and existing development status of positive psychology. It is argued that the principles of positive psychology have surfaced in prior psychological research, after a thorough introduction and analysis (Li, 2019). Although it is not brand new, Vickers (2018) and Fu and Clarke (2019) believe that its systematic and integrated study can give a new research direction for psychology. In “Positive Psychology: Ideas and Actions,” Miao Yuanjiang presented the core framework material of positive psychology as an idea and an action (Yuen et al., 2019). It describes the massive reaction it has elicited in the academic and social areas, and it looks ahead to the positive psychology’s future growth trend with both optimism and caution (Tao, 2017; Huang et al., 2021). Grit is a culture of learning that encourages students to persevere through a succession of setbacks until they achieve success. Cultivation grit is a crucial personality trait that China’s modern school system must emphasize in its program. When doing educational reform research, it is important to consider the impact of this personality trait on educational outcomes.

This study intends to promote educational curriculum reform, as well as research and evaluate design education curriculum reform based on positive psychology, as well as the weaknesses and defects of traditional design curriculum education. Examine and debate the design of education curriculum reform based on the idea of positive psychology.

## Applied Research Based on Positive Psychology-Oriented Design and Education Curriculum Reform

### Basic Principles of Education Curriculum Reform

#### Emphasize the Combination of Classroom Teaching and Practical Work

The design course considers a curriculum development model on the basis of the work process, integrates classroom teaching and actual work processes, advances knowledge, technology, and crafts directly regarding the work process, incorporates real design topics into classroom teaching, and simulates topics to enhance the teaching effects (Rosenbaum, 2018; Yang et al., 2020). This course, on the other hand, closely integrates classroom instruction and social practice (practice), arranges students to practice internships, and requires students to spend their summer vacation at design firms, printing firms, and graphic production (plate manufacturing) firms. We should maintain the original qualities of the design curriculum and highlight the educational style of integrating academic education and practical education as we change the design curriculum.

#### Emphasize the Interaction Between Theory and Practice

The design course focuses on the specificity and coherence of knowledge. In addition, the design curriculum puts a certain emphasis on knowledge application. A novel visual communication design teaching technique that integrates theory and practice is produced *via* the analysis and induction of knowledge points and practical teaching activities. This educational model is distinct from basic education and emphasizes the teachers’ motivation and perseverance in the course. Encourage students to put their theoretical knowledge, into practice, make mistakes in practice and write a summary of their experience. In this process, the student’s grit personality is extremely crucial. For instance, the connection between the image discreteness theory and the quality of printing and digital display of image restoration. Another example is the use of digital network technology, which necessitates a cooperative relationship between theory and practice in order to produce optimal results. In the case of initially acquired theoretical knowledge, none of these practical acts can be completed. Students’ skills are gradually improved through cross-teaching of theory and practice. As a result, in design educational reform, the connection between theory and practice should be highlighted, and the relationship between theory and practice may be leveraged to improve design outputs.

#### Emphasize the Organic Connection Between Basic and Professional Applications

The design course demonstrates how fundamental and professional applications are inextricably linked. During Zhu’s design education on T-structure knowledge points in industrial design, T represents the organic relationship between fundamental and professional application in the structure and highlights this in teaching content, methodologies, and other aspects (Shen et al., 2019; Julie 2020). As a result, the design course focuses on the teaching and training of fundamental high-intensity design knowledge and skills, as well as thorough design application training. Aside from that, there was a lot of discussion in other design classes on the link between basic and professional apps. For instance, in a visual communication design course for point-line-plane knowledge points, we allow students to generate basic colorful drawings, such as theoretical knowledge, i.e., fundamental applications. After a student has a thorough understanding of the point of knowledge, the teacher will steer the student toward professional application, such as considering Knowledge to design posters, packaging, and other materials.

#### Emphasize the Cultivation of Grit in the Modern Education System

This course focuses on cultivating grit, a crucial personality trait that students, particularly those in colleges and institutions, must master in order to succeed in school and the future job market. Cultivation grit is a scenario, where a person perseveres through several challenges without getting the desired result and never gives up until eventual success is achieved. This is a potential personality that education aims to develop among today’s students, especially in college. The main objective of this course is to equip the learners with strong cultivation grit skills, so that they may build on their strong theoretical foundation and stay motivated until they achieve their goals without giving up. Several studies have found that cultivating grit can improve educational performance. As a result, the psychological and physical conditions of humans at a variety of age groups suggest that the grit-oriented character of education may be successfully adopted in the college educational system to equip students with the soft skill of surviving adversity. Because upgraded design education aims to prepare learners for the future, which requires a great deal of perseverance, motivation, and persistence. Thus, it is not only urgent but also important to have an education system that promotes natural programs with an aim to enhance the cultivation of grit among college and university students.

### The Role of Positive Psychology in Promoting Curriculum Education Reform

Curriculum school reform concepts have existed in China from ancient times. Positive psychology, on the other hand, provides theoretical direction for the new curriculum reform, while the new curriculum reform encourages the growth of positive psychology in China. This is not the first time that positive psychology has arisen in the Chinese educational policies since the creation of New China. Positive psychology focuses on one central topic (happiness), as well as three essential points (emotional experience, personality traits, and organizational system). Its primary educational philosophy is to stick to the goal of academic growth and personality integrity, consider and assess the students positively, lead and achieve students positively, and make each student the best version of himself. Active education promotes the concept of “achieving every student’s happy growth and becoming a talented person,” emphasizing the need of boosting students’ good life experiences by assisting them in cultivating a positive mind, cultivating positive character, and realizing a positive existence. Owing to positive psychology, our country’s educational paradigm shifted from transferring knowledge to nurturing students’ cooperative learning and creativity. Second, education has shifted from a teacher-centered to a student-centered approach. Students should give their subjective status and role full play in class so that they may actively engage in teaching, develop their lively growth, and build their creative psychological quality. Finally, teachers must enhance their creativity. Based on effective knowledge transfer and learning, we should foster the ability to realize the knowledge, technology, and management innovations, highlighting the process of identifying knowledge, resolving the stress problem, and developing the students’ exploration spirit. This shift has resulted in a gradual transition in China’s educational system from cramming to addressing students’ requirements. Therefore, positive psychology took root in China prior to the new curriculum reform and has since increased in popularity. The introduction of new curriculum reform is undoubtedly a catalyst, accelerating the development of curriculum education reform.

### Analysis of Problems in Modern Education Curriculum Reform

In China, educational development has been shaped by decades of reform and adjustment, yielding a series of successes that have supported the country’s social development, economic growth, and competency enhancement. However, some of its issues have impeded educational progress and caused a series of social issues that have resulted in concealed societal issues. (Hong and Liu, 2019; Wei, 2019). The first is the inequity of people’s entitlement to education, which varies by area, occupation, ethnicity, and income, with limited quality educational resources skewed heavily toward the wealthy and powerful. Second, the problem with excessive industrialization of education is that it has transferred educational concepts to parents with the goal of educational progress, speed, and economic efficiency. Note: there are a variety of anomalous fees at schools at all levels. The dominance of cramming and problem-solving teaching approaches have resulted in the dominant mode, which has formed the issue of academic frivolity. Third, education reform is highly political, the policy cannot be adjusted to local conditions or individual situations, lack of accountability mechanisms, and face-saving schemes are frequent. Finally, under the entrance examination education system, students receive points. The supreme mind is unable to explore and think independently. Students’ academic burden is weighed, and their physical and mental health, as well as their personal growth, are not assured. For example, the rate of visual impairment among elementary, middle, and high school students improved by 10.4, 8.8, 11.5, and 3.3 percentage points, respectively, in comparison with 1995. The average lung capacity of male and female students aged 7–18 declined by 206 ml and 122 ml respectively, when compared to 2000. Professor Zhao Yong of Michigan State University has also highlighted several misconceptions regarding education reform with regard to learning from other countries: First, do we spill our children and bathwater together, forget about our still useful experiences, and overlook our own strengths? Second, we only look at the positive features of others’ work while ignoring their shortcomings, thirdly, we overlook the circumstances and environment in which others succeed, and fourth, we examine each effective technique and practice in isolation. A brief explanation of how each success factor can be combined to generate the desired impact; Fifth, we are judging other nations’ experiences only through our own eyes, which inevitably leads to some misunderstanding. (Shi, 1985) The educational growth of China has various challenges as a result of the reform of the public education system, particularly following the start of the industrialization of education. In this context, active positive psychology intervention is quite significant in educational reform since it may boost students’ and instructors in stimulating students’ and teachers’ motivation. Positive psychology promotes whole personality growth and positive energy exploration, which may completely excite students’ passion and shift education away from information transfer and toward stimulating student collaboration and invention. For instance, in the design lesson, students will participate in sitcom simulation and re-enact their daily lives. Through reflection, we may better understand ourselves, explore our potentials, and realize the value of life.

Assessing the effectiveness of educational reforms, on the other hand, is also a source of concern. The achievements of educational reform include the generalization of the concept of educational reform, the pursuit of performance, the lack of actual third-party review, and the improvement of certain students’ test results. It is irrational to consider the external development accomplishments of education reform as achievements of education reform, to overstate the achievements of education reform, and to depart from the ethical basis of education reform in today’s appraisal of education reform. In light of this, it is particularly vital to rely on advanced international experiences (Zhong and Zhou, 2019). In 2006, the INQAA HE, an international organization for quality protection of higher education, explained in its evaluation rules that universities are largely responsible to ensure and improve the quality of higher education (INQAAH, 2007). In the United Kingdom, higher education secures the students’ interests by higher academic awarding criteria, certification in the US safeguards educational interests, and the Japanese education system protects the students’ rights through frequent university self-evaluation. Despite the variances in assessment methodologies, nations such as the United Kingdom, South Korea, and Australia provide funding to schools based on evaluation results, which are publicly revealed and reviewed on a regular basis. Inspired by the international evaluation system, China’s educational reform assessment must be first, perfect the index system, and improve the measures to ensure the quality of colleges and universities. Second, information is quite transparent, with worldwide traffic norms mentioned to guarantee that information is real-time, accurate, and open. Third, devolve power, exercise subjective initiative, enhance self-innovation, adapt to local conditions, refer to worldwide practice, and promote management subject and enforcement to help university move from” do evaluation” to” control evaluation.” A reformulation in which the subject of a row is physically separated.

### Design Education Curriculum Reform Strategy Based on Positive Psychology Orientation

The positive psychology-based design education curriculum change is progressing steadily. Implementing the new round of basic education curriculum reform has promoted various gratifying changes to school education and teaching and higher requirements for teachers. At the same time, higher standards for the level of talents fostered by upper normal institutions are being imposed (Lomas and Ivtzan, 2016; Mercer, 2017). Many ordinary colleges are falling behind in dealing with this transition as compared to the orderly implementation of curriculum reform in basic education. They have not improved educational principles, teaching concepts, teaching techniques, and curriculum structure. For instance, in teaching, there is a single objective, and instruction is primarily controlled by the principle of students’ mastery of the syllabus norm, but the teaching process does not focus on the healthy growth and overall development of students. Although the prior form of education is no longer cramming and case teaching techniques are adopted to improve the emotional contact between instructors and students, the teaching mode lags. Teachers cannot effectively enhance the students’ enthusiasm in short lessons. The teaching content is negative, and the teacher is unable to provide constructive feedback to students on issues, such as repeated mistakes in design education and the smooth development of teaching.

#### Change Education Concept

Teachers have a critical role in educational reform, which is a necessary component of enhancing teaching quality. In order to properly activate students’ subjective initiative and creativity, teachers must adapt their educational concepts in the educational process, prioritize positive psychology, and accept positive psychology as the leading role. Second, teachers must have specific innovative ideas and innovative consciousness, focus on cultivating the ability of knowledge innovation, actively respond to difficulties in the classroom, and create answers throughout the teaching practice. Innovative thinking is very crucial in design education, and it is also a key component of positive psychology research. Innovation allows not just intellectual but also non-intellectual factors, which are critical to creative activities, not only in terms of stimulating creative awareness but also promoting creative thinking for better play and usage. For instance, teachers must be able to swiftly move from conveying information to nurturing students’ ability to create freely in the brainstorming instruction of the visual design creative thinking course. Secondly, it is necessary to provide positive guidance to students, focus on students, and give students positive affirmation. This can promote the students’ creativity and allow them to better complete their tasks.

#### Use Creative Teaching Methods

Designing creative teaching techniques in classrooms refers to getting half of the rules of teaching in teaching activities, promoting students’ positive attitudes, and using their own creative thinking to investigate information.

For instance: in the impromptu demonstration technique, in the design class, the students act as the initial party to improvise the customer demand for the teacher, and the teacher performs a mock-up. Students may see the entire process of the teacher’s creative thought as well as the entire process of the project operation during the teacher’s demonstration, and gain benefit and inspiration from the observation. In the teaching method of incomplete content, in the design teaching, the teacher does not share all of the knowledge in its entirety, but consciously forms a blank in the teaching content, allowing students to develop their own conclusions. This can encourage students’ desire for exploration and innovative thinking ability; the situational experience method promotes the project benchmarking meeting in the design classroom, allowing students to immerse themselves in the situation, fully express their creativity, actively resolve the problems faced in the meeting, continue to grow in the face of obstacles, and enhance the knowledge system. Simultaneously, it is part of the instructional process to encourage students’ perseverance and boost their personality development.

#### Create a Suitable Environment for “Active School Education”

In order to fulfil educational goals, we must priories the building of an appropriate educational environment, particularly a soft environment. Care, trust, and respect for diversity are vital value factors in the building of a soft environment, goals, plans, and motivation are critical process elements; hope and social contribution are complete result aspects. Together, the three contribute to happiness. A learning environment that is both meaningful and suitable. At the same time, teachers should develop educational goals and instructional strategies based on their care and respect for students, develop educational goals and teaching plans.

The program’s integrated design and students’ learning motivation can help students’ core literacy “come from the situation” and “get to the situation” more effectively. The construction of an integrated learning field of value, process, and result based on positive school education in the teaching reform of basic education in the new era is a new way of thinking about using positive psychology to create a teaching environment in the process of education and teaching activities. Building a positive educational environment is critical to construct the teaching objective, with an aim to cultivate psychological quality. Such teaching objectives can initially stimulate the students’ self-confidence. They feel a sense of achievement and satisfaction while completing tasks, and develop a positive sense of self-efficacy; secondly, it can build optimism in students. Students can enjoy the fun of Wanheng goals and develop a positive attitude toward learning by dividing the design time into small goals in stages and formulating action plans to implement them; and third, it can cultivate students’ resilience by guiding students to correctly identify the gap between ideal and display, correctly handle the setbacks, and face challenges with a positive attitude of problem-solving.

## Experimental Research Based on Positive Psychology-Oriented Design Education Curriculum Reform

### Subjects

In order to investigate whether the design education curriculum reform based on the direction of positive psychology has improved feasibility than the conventional education curriculum reform and whether cultivating students’ grit plays a dynamic role in the curriculum reform of design education based on the direction of positive psychology, I conducted a questionnaire. In the research object of this research for Chinese design students who have taken positive psychology-oriented courses and those who have not, the questionnaires were divided into two groups: the psychology-based design education course and the conventional teaching courses. The survey content has a totality of 20 items. Among them, the experience of the education course consists of 10 items and the role of educational curriculum reform consists of 10 items, all of which are derived from mature scales and modified according to actual research needs. The survey method is mostly based on the online questionnaire survey. Questionnaires will be disseminated and gathered from March 4th to March 10th, 2022. A total of 230 questionnaires were distributed and 200 suitable questionnaires were recovered. The effective recovery rate was 86.96%, of which, there are 100 design education courses based on the direction of positive psychology, and 100 traditional teaching courses.

### Research Methods

In order to make certain of the scientificity of the research results, the returned questionnaires were screened and cleaned, and it was found that the time to fill in the questionnaires was not long enough and the answers had certain regularity. Invalid questionnaires were quantitatively described and analyzed utilizing SPSS and other sociological statistical processes, and the autonomous sample *t*-test was particularly used to test the dissimilarity of diverse contents between the two groups taking part in the positive psychology-based design education course and the traditional teaching course. The mean standard deviation, t value, and significance were used for comparative analysis.

### Reliability and Validity Analysis

#### Reliability Analysis

Cronbach’s Alpha Coefficient of Concordance (α) was used to test the reliability and validity of questionnaire-based measures. It is deemed that if the coefficient α is greater than 0.7, the dependability of the questionnaire is high. SPSS statistical software package has been used to process the data, as shown in Table 1, the general alpha coefficient of all items in this questionnaire is 0.764, signifying that the questionnaire is dependable and reliable in terms of internal consistency.

**Table 1:** Reliability analysis results.

Cronbach’s alpha	Cronbach’s alpha based on standardized terms	Items
0.744	0.764	20

#### Validity Analysis

The integrity of the questionnaire is the most significant trait of the questionnaire survey, and the aim of the survey is to acquire high integrity and accurate conclusions; the higher the validity of the questionnaire, the more genuine the survey behavior. The questionnaire survey makes it easier to attain the aim of high validity, and the questionnaire will be accurate and effective. The validity test results are revealed in Table 2. Since all items are derived from mature scales and adjusted according to authentic research needs, they have certain content validity, and the validity of the scale is tested; the KMO value is 0.726 > 0.7, Bartlett’s sphericity test approximate card. The square is 5512.13, the df degree of freedom is 190, and the significance is 0.000, indicating good validity.

**Table 2:** The results of the difference analysis of the educational curriculum feelings of participants in the educational curriculum reform in the direction of positive psychology and the traditional educational curriculum reform.

	Types of educational courses	*N*	Mean	SD	*t*	Sig.
1	Positive psychology orientation education course	100	4.52	0.969	2.165	0.032
	Traditional education courses	100	4.22	0.991		
2	Positive psychology orientation education course	100	4.25	0.914	1.305	0.194
	Traditional education courses	100	4.08	0.929		
3	Positive psychology orientation education course	100	4.36	0.871	2.750	0.007
	Traditional education courses	100	4.05	0.716		
4	Positive psychology orientation education course	100	4.07	0.977	4.281	0.000
	Traditional education courses	100	3.57	0.640		
5	Positive psychology orientation education course	100	4.06	1.013	3.272	0.001
	Traditional education courses	100	3.60	0.974		
6	Positive psychology orientation education course	100	4.13	0.861	5.792	0.000
	Traditional education courses	100	3.53	0.577		
7	Positive psychology orientation education course	100	4.44	0.925	1.260	0.209
	Traditional education courses	100	4.27	0.983		
8	Positive psychology orientation education course	100	4.46	0.858	1.883	0.061
	Traditional education courses	100	4.20	1.082		
9	Positive psychology orientation education course	100	4.36	0.990	1.623	0.106
	Traditional education courses	100	4.11	1.180		
10	Positive psychology orientation education course	100	4.32	0.952	2.129	0.035
	Traditional education courses	100	4.00	1.163		
Education course experience	Positive psychology orientation education course	100	4.30	0.407	6.013	0.000
	Traditional education courses	100	3.96	0.378		

## Experimental Analysis of Design Education Curriculum Reform Based on Positive Psychology Orientation

### Analysis of Dissimilarities in Educational Curriculum Opinions Between the Positive Psychology Educational Curriculum Reform and the Conventional Educational Curriculum Reform

Table 2 reveals the dissimilarities in the educational curriculum opinions of the students who participate in the positive psychology-oriented educational curriculum reform and the conventional educational curriculum reform. In this study, the autonomous sample t-test was utilized to explore. From Table 1, it can be seen that there is a considerable difference between the students taking part in the positive psychology education curriculum reform and the conventional education curriculum reform in the experience of the education curriculum (*p* < 0.05). “It enables me to better understand the hypothesis, so as to combine design theory with design practice.” “The educational reform using positive psychology in design education will activate the classroom atmosphere, and use ‘empathy’ psychological empathy to increase the distance between teachers and students.” There was no noteworthy difference (*p* > 0.05). Specifically, the educational curriculum experience of students taking part in the positive psychology education curriculum reform is larger than that of students taking part in the traditional education curriculum reform, signifying that there are dissimilarities in the educational curriculum experience of students taking part in different educational curriculum reforms, and the design of positive psychology-oriented educational curriculum. The reform has better feasibility than the conventional education curriculum reform.

**Table 3:** Difference analysis results of students’ grit feelings in different educational courses.

	Types of educational courses	*N*	Mean	SD	*t*	Sig.
11	Positive psychology orientation education course	100	4.36	0.990	1.166	0.245
	Traditional education courses	100	4.19	1.070		
12	Positive psychology orientation education course	100	4.14	1.119	1.275	0.204
	Traditional education courses	100	3.93	1.208		
13	Positive psychology orientation education course	100	4.37	0.872	2.836	0.005
	Traditional education courses	100	4.05	0.716		
14	Positive psychology orientation education course	100	4.49	0.745	1.428	0.155
	Traditional education courses	100	4.36	0.523		
15	Positive psychology orientation education course	100	4.10	0.990	4.301	0.000
	Traditional education courses	100	3.59	0.653		
16	Positive psychology orientation education course	100	4.07	1.018	3.336	0.001
	Traditional education courses	100	3.60	0.974		
17	Positive psychology orientation education course	100	4.16	0.873	6.022	0.000
	Traditional education courses	100	3.53	0.577		
18	Positive psychology orientation education course	100	4.25	0.914	1.073	0.285
	Traditional education courses	100	4.11	0.931		
19	Positive psychology orientation education course	100	3.84	1.170	0.122	0.903
	Traditional education courses	100	3.82	1.158		
20	Positive psychology orientation education course	100	4.39	0.815	1.026	0.306
	Traditional education courses	100	4.27	0.839		
The role of grit	Positive psychology orientation education course	100	4.217	0.445	4.748	0.000	
	Traditional education courses	100	3.945	0.361		

### Difference Analysis of the Feelings of Students’ Grit in Positive Psychology Education Courses and Traditional Education Courses

Table 3 shows the dissimilarities in the awareness of students’ grit in positive psychology-oriented education courses and conventional education courses. In this study, the autonomous sample *t*-test was utilized to examine. It can be seen from Table 4 that the role of grit in dissimilar educational curriculum reforms is appreciably different (*p* < 0.05). “The training of the design has enabled me to have a clear goal when designing, and I am no longer blind.”; “The training of perseverance enables me to act proactively and not give up when I encounter problems in design.”; and “The training of perseverance has strengthened me. There are also significant differences in items such as my planning and execution of the design project.” Specifically, the role of grit in taking part in the educational curriculum reform oriented to positive psychology is considerably higher than that in the conventional educational curriculum reform, signifying that grit has not only made a difference in cultivating students’ feelings in different educational curriculum reforms, but it is based on the orientation of positive psychology. There is a more noticeable positive role in the reform of the design education curriculum.

**Table 4:** Validity analysis results.

Kaiser-Meyer-Olkin metric for sampling adequacy		0.726
Bartlett’s test for sphericity	Approximate chi-square	5512.130
	df	190
	Sig.	0.000

## Conclusion

By analyzing the current problems in design education, this paper draws out the importance of design education reform and puts forward the concept of design education reform guided by positive psychology. First, in the theoretical part, the paper discusses the positive psychology-oriented design education reform from four parts. The first part proposes three principles for the positive psychology-oriented design curriculum reform. First, Emphasize the combination of classroom teaching and practical work; second, Emphasize the interaction between theory and practice; third, Emphasize the organic connection between basic and professional applications; and fourth, Emphasize the Cultivation of grit in the modern education system. The second part discusses the role of positive psychology in promoting curriculum education reform by analyzing the process of China’s education reform. The research results show that the application of positive psychology in teaching reform can improve students’ creativity, stimulate students’ subjective initiative, and cultivate students’ enthusiasm. Accelerating the transformation of China’s education system from cramming to meeting students’ needs is a catalyst for education reform. The third part analyzes the basic problems faced in modern China’s educational reform, and how to improve the evaluation system of educational reform. The last part proposes the strategies of designing curriculum reform based on positive psychology. First, Change the education concept; Second, Use creative teaching methods; and Third, Create a suitable environment for “active school education.”

Second, in order to investigate whether the design education curriculum reform based on the direction of positive psychology has improved feasibility than the conventional education curriculum reform and whether cultivating students’ grit plays a dynamic role in the curriculum reform of design education based on the direction of positive psychology, I conducted a questionnaire. By analyzing the results of the questionnaire through SPSS, I found that the positive psychology-oriented design course is more feasible than the traditional design course, and the cultivation of grit has a positive effect on the positive psychology-oriented course, making the reform more effective.

Positive psychology-oriented design education reform can give full play to students’ subjective initiative and stimulate students’ creativity. Grit training enhances students’ problem-solving ability and grit in learning in the positive psychology-oriented design education reform. When doing sample interviews, I found that the number of students receiving positive psychology-oriented design education is small, and the duration of this education is short. Therefore, positive psychology-oriented design education reform should be implemented on a large scale.

## Data Availability Statement

The original contributions presented in the study are included in the article/supplementary material, further inquiries can be directed to the corresponding author.

## Ethics Statement

Ethical review and approval was not required for the study on human participants in accordance with the local legislation and institutional requirements. Written informed consent from the (patients/participants or patients/participants legal guardian/next of kin) was not required to participate in this study in accordance with the national legislation and the institutional requirements.

## Author Contributions

YW: statistical data analyses and writing of the manuscript. KK: critical review of the manuscript. All authors contributed to the article and approved the submitted version.

## Conflict of Interest

The authors declare that the research was conducted in the absence of any commercial or financial relationships that could be construed as a potential conflict of interest.

## Publisher’s Note

All claims expressed in this article are solely those of the authors and do not necessarily represent those of their affiliated organizations, or those of the publisher, the editors and the reviewers. Any product that may be evaluated in this article, or claim that may be made by its manufacturer, is not guaranteed or endorsed by the publisher.
